# Phytotoxic Effects of Plant Essential Oils: A Systematic Review and Structure-Activity Relationship Based on Chemometric Analyses

**DOI:** 10.3390/plants10010036

**Published:** 2020-12-25

**Authors:** Ahmed M. Abd-ElGawad, Abd El-Nasser G. El Gendy, Abdulaziz M. Assaeed, Saud L. Al-Rowaily, Abdullah S. Alharthi, Tarik A. Mohamed, Mahmoud I. Nassar, Yaser H. Dewir, Abdelsamed I. Elshamy

**Affiliations:** 1 Plant Production Department, College of Food and Agriculture Sciences, King Saud University, P.O. Box 2460, Riyadh 11451, Saudi Arabia; assaeed@ksu.edu.sa (A.M.A.); srowaily@ksu.edu.sa (S.L.A.-R.); 437105762@ksu.edu.sa (A.S.A.); ydewir@ksu.edu.sa (Y.H.D.); 2 Department of Botany, Faculty of Science, Mansoura University, Mansoura 35516, Egypt; 3 Medicinal and Aromatic Plants Research Department, National Research Centre, Cairo 11865, Egypt; aggundy_5@yahoo.com; 4 Chemistry of Medicinal Plants Department, National Research Centre, 33 El-Bohouth St., Dokki, Giza 12622, Egypt; ta.mourad@nrc.sci.eg; 5 Chemistry of Natural Compounds Department, National Research Centre, 33 El Bohouth St., Dokki, Giza 12622, Egypt; mnassar_eg@yahoo.com (M.I.N.); ai.el-shamy@nrc.sci.eg (A.I.E.); 6 Department of Horticulture, Faculty of Agriculture, Kafrelsheikh University, Kafr El-Sheikh 33516, Egypt; 7 Faculty of Pharmaceutical Sciences, Tokushima Bunri University, Yamashiro-Cho, Tokushima 770-8514, Japan

**Keywords:** allelopathy, bioherbicides, volatile oils, terpenes, aromatic plants

## Abstract

Herbicides are natural or synthetic chemicals used to control unwanted plants (weeds). To avoid the harmful effects of synthetic herbicides, considerable effort has been devoted to finding alternative products derived from natural sources. Essential oils (EOs) from aromatic plants are auspicious source of bioherbicides. This review discusses phytotoxic EOs and their chemical compositions as reported from 1972 to 2020. Using chemometric analysis, we attempt to build a structure-activity relationship between phytotoxicity and EO chemical composition. Data analysis reveals that oxygenated terpenes, and mono- and sesquiterpenes, in particular, play principal roles in the phytotoxicity of EOs. Pinene, 1,8 cineole, linalool, and carvacrol are the most effective monoterpenes, with significant phytotoxicity evident in the EOs of many plants. Caryophyllene and its derivatives, including germacrene, spathulenol, and hexahydrofarnesyl acetone, are the most effective sesquiterpenes. EOs rich in iridoids (non-terpene compounds) also exhibit allelopathic activity. Further studies are recommended to evaluate the phytotoxic activity of these compounds in pure forms, determine their activity in the field, evaluate their safety, and assess their modes of action.

## 1. Introduction

Humans have been cultivating plants for nearly 10,000 years ago. Today, any plant growing where it is not wanted is defined as a weed. Weeds represent an important constraint to agricultural production [1]. Weeds represent approximately 0.1% of the world’s flora and they evolve with agricultural practices. Weeds can cause declines in crop yields via competition for resources such as light, water, space, and nutrients, and by producing chemical weapons known as allelopathic compounds [2]. Weed management is achieved using several techniques to limit infestation and minimize competition. These techniques evolved to mitigate crop yield losses, but weed control is typically used only after a problem has been identified.

Scientists and researchers address weed control through physical, chemical, and biological methods. Controlling weeds in an environmentally friendly way is often considered a challenge. Natural resources offer new approaches to producing eco-friendly, and safe bioherbicides that are effective against nuisance weeds. Plants produce the essential oils (EOs) in their various organs as a complex mixture of secondary metabolites such as mono-, sesquit-, and di-terpenoids in addition to hydrocarbons [3,4]. In plants, EOs were biosynthesized via different isoprenoid pathways such as methylerythritol phosphate (MEP) pathway and mevalonic acid (MVA) pathway [5]. The EOs have been described as potent biological agents such as phytotoxic [6,7,8,9], antimicrobial [10], anti-inflammatory, antipyretic [11], antiulcer [12], and hepatoprotective [13]. The bioactivities potential of EOs are directly correlated with the quality and quantity of their chemical constituents [6]. Many studies have been performed using the extracted EOs from various plants as phytotoxic chemicals (allelochemicals), where the phytotoxicity is usually correlated to the whole EO profile that contained a mixture of compounds. However, the activity of the EO could be ascribed to a specific compound(s) in the EO. Therefore, in the present review, we try to elucidate a framework of the most frequent and major allelochemicals that were identified in the EOs with a substantial phytotoxic activity using chemometric tools. Additionally, the activities of the authentic identified major compounds are discussed.

## 2. Materials and Methods

This review focuses on reports of EOs from plants that exhibit phytotoxic activity published between 1972 and early 2020, using Google, Sci-finder, Google Scholar, PubMed, Elsevier, and Springer databases. Based on the major compounds (those constituting > 5% of the total mass of the EO), the plants were categorized into three groups; mono-, sesqui-, and non-terpenoid–rich compounds. Firstly, the database of EOs rich in monoterpenes derived from plants comprised 46 species belonging to 12 botanical families, including Lamiaceae (18 species), Myrtaceae (nine species), Asteraceae (eight species), Anacardiaceae (three species), and Cannabaceae, Euphorbiaceae, Monimiaceae, Pinaceae, Poaceae, Verbenaceae, Winteraceae, and Apiaceae (a single species each). Additionally, the EOs of these plants were tested against 49 plant species.

Secondly, the plant EOs rich in sesquiterpenes from 25 plant species belonging to eight botanical families were studied. The most represented botanical families were Lamiaceae and Asteraceae (nine plant species each), while Anacardiaceae, Boraginaceae, Fabaceae, Myrtaceae, Simaroubaceae, Verbenaceae, and Chenopodiaceae were represented by a single species. All the EOs of these plants were investigated against 13 plant species. Thirdly, six plant EOs rich in non-terpenoid compounds were identified belonging to Lamiaceae (three species), Apiaceae (two), and Cucurbitaceae (one), were tested against 17 plants. 

To assess the correlation of EOs phytotoxic activity and structural compounds, a data matrix of each group was performed as a spreadsheet in MS-EXCEL. A matrix of 42 major monoterpene compounds from 45 plant species was assembled, while a matrix of 26 sesquiterpene compounds, identified in the EOs of 22 plant species was prepared. These matrices were subjected to PCA using XLSTAT software version 14 (Addinsoft, New York, NY, USA). 

## 3. Phytotoxic EOs Derived from Plants Rich in Monoterpenes

The EOs from different plant species with monoterpenes as the main compounds that exhibited significant phytotoxic activity against various target plant species are presented in Table 1. Zhang, et al. [14] concluded that monoterpene-rich EOs derived from *Eucalyptus salubris*, *E. dundasii*, *E. spathulata*, and *E. brockwayii* strongly inhibited germination and seedling growth in *Solanum elaeagnifolium* relative to commercial Eucalyptus oil and 1,8-cineole. Moreover, the EO of *E. salubris* was found to be the most powerful inhibitor of germination and roots and shoot growth, while *E. spathulata* exhibited the lowest effect [14].

Hydro-distilled EO from *Senecio amplexicaulis* with a high content of monoterpenes, including *α*-phellandrene, *O*-cymene, and *β*-ocimene, reportedly exhibited strong allelopathic activity at higher concentrations, with a significant ability to inhibit germination of *Phalaris minor* and *Triticum aestivum* seeds [47]. The EOs of *Heterothalamus psiadioides*, composed mainly of the monoterpenes *β*-pinene, Δ3-carene, and limonene, showed cidal effects against *Lactuca sativa* and *Allium cepa* by inhibiting germination as well as growth of shoots and roots [29]. Moreover, a strong herbicidal activity against *Amaranthus retroflexus*, *Chenopodium album*, and *Rumex crispus* was reported in the EOs of the two *Tanacetum* species (*T. aucheranum* and *T. chiliophyllum*) by completely inhibiting seed germination and seedling growth, an ability that may be attributable to their monoterpene content, including 1,8-cineole, camphor, borneol, and terpinen-4-ol [46]. Significant reduction of seedling emergence and growth of *Sinapis arvensis*, *Diplotaxis harra*, *Trifolium campestre*, *Desmazeria rigida*, and *Phalaris canariensis* were reported via the EO derived from *E. lehmanii* in which monoterpenes represented the major constituents, including 1,8-cineole, *α*-thujene, and *α*-pinene [45].

The EOs from *Agastache rugosa* leaves collected over different seasons reportedly achieved partial or complete prevention of germination and growth of hypocotyl and radicles in *Majorana hortensis*, *Trifolium repens*, *Rudbeckia hirta*, *Chrysanthemum zawadskii*, *Melissa officinalis*, *Taraxacum platycarpum*, and *Tagetes patula*. These extracted EOs were described to be rich in the monoterpenes methylchavicol, *d*-limonene, and linalool as the main compounds [44]. In the same manner, the chemical profiles of the EOs of Chilean *Peumus boldus* and *Drimys winterii* were reported to be composed primarily of the monoterpenes ascaridole, *p*-cymene, and 1,8-cineole and *γ*-eudesmol, elemol, and terpinen-4-ol. These two EOs were found to exhibit inhibitory effects against *Amaranthus hybridus* and *Portulaca oleracea* [43]. The EO of *Peumus boldus* was found to inhibit germination and seedling growth in two weeds at all used concentrations, while *Drimys winterii* EO exhibited inhibitory activity against germination activity in *Portulaca oleracea* only at the highest dose (1 μL mL^−1^) [43]. In addition, de Oliveira, et al. [42] reported that different samples of EOs extracted by supercritical CO_2_ from *Syzygium aromaticum* at varying temperatures and pressures displayed allelopathic activities by inhibiting germination and radicle elongation in *Mimosa pudica* and *Senna obtusifolia*, with extraction of the EO at 50 °C and 300 bars associated with the most effective activity. The monoterpenes eugenol, eugenol acetate, and (E)-caryophyllene were reported as the main constituents of these samples. The EOs of the leaves and fruits of *Shinus molle* were reported to cause a concentration-dependent decline in the germination and radicle elongation of *Triticum aestivum* with more activity seen in leafy samples. Both oils were found to be composed of monoterpenes as the main components, with an abundance of *β*-phellendrene, *α*-phellendrene, and myrcene [41].

The chemical components as well as the phytotoxic activities of EOs derived from 12 Mediterranean plants, including *Hyssopus officinalis*, *Lavandula angustifolia*, *Ocimum basilicum*, *Majorana hortensis*, *Origanum vulgare*, *Salvia officinalis*, *Foeniculum vulgare*, *Thymus vulgaris*, *Melissa officinalis*, *Verbena officinalis*, *Pimpinella anisum*, and *Carum carvi*, on germination and radicle growth in *Raphanus sativus*, *Lactuca sativa*, and *Lepidium sativum* seeds have been documented [40]. The EOs reportedly have an inhibitory effect against germination and initial radicle elongation at different doses through different mechanisms, with samples of *Melissa officinalis*, *Thymus vulgaris*, *Verbena officinalis*, and *Carum carvi* demonstrating the strongest effect. Monoterpenes were described as the main components of *Hyssopus officinalis*, *Lavandula angustifolia*, *Majorana hortensis*, *Melissa officinalis*, *Ocimum basilicum*, *Origanum vulgare*, *Salvia officinalis*, *Thymusvulgaris*, *Verbena officinalis*, and *Carum carvi*, while non-terpenoid phenols were the main constituents in *Foeniculum vulgare* and *Pimpinella anisum* [40]. Similarly, peppermint EO is reportedly rich in menthone, menthol, and menthofuran, and has been described as having a potent allelopathic effect on seed germination and seedling growth in *Lycopersicon esculentum*, *Raphanus sativus*, *Convolvulus arvensis*, *Portulaca oleracea*, and *Echinochloa colonum* [39]. In 2010, the main components of *Zataria multiflora* EO were reported as the monoterpenes carvacrol, linalool, and *p*-cymene, all of which exhibited significant herbicidal activities against *Hordeum spontaneum*, *Secale cereal*, *Amaranthus retroflexus*, and *Cynodon dactylon* [38]. This activity was associated with significant inhibition of the rate of germination, seedling length, root and stem fresh and dry weights at all used concentrations, and 320 and 640 mL L^−1^ in particular [38].

The dose-dependent toxicity of the EOs of *Cotinus coggyria*, which consist mainly of monoterpenes such as limonene, *α*-pinene, and *β*-myrcene, against the weeds *of Silybum marianum* and *Portulaca oleracea* reportedly [37] decreased germination in radishes by 83% and 60%, seedling radicle length by 93% and 84%, and plumule length by 84% and 91% at 32 µL mL^−1^. The EOs of *Pinus brutia* and *Pinus pinea* were documented to have monoterpenes as the major components, with a preponderance of *α*- and *β*-pinene and caryophyllene [35]. At higher dose, these EOs were found to have a potent inhibitory effect on germination by 53% and 22% of *Lactuca sativa*, 60% and 33% of *Lepidium sativum*, and 13% and 3% of *Portulaca oleracea*, respectively [35]. An evaluation of Tunisian *Pinus pinea* EO, rich in limonene, *α*- and *β*-pinene, revealed a dose-related gradual inhibition of *Lolium rigidum*, *Sinapis arvensis*, and *Raphanus raphanistrum*, and seed germination was completely inhibited at low concentrations [36]. The EO of *Plectranthus amboinicus*, which is composed primarily of monoterpenes, and carvacrol in particular (88.61%), was reported to significantly inhibit germination and reduce the growth of *Lactuca sativa* and *Sorghum bicolor* roots and shoots [34]. Another study [33] described the EO chemical composition of two Eucalyptus plants in which monoterpenes, including p-cymene, *β*-myrcene, and (+)-limonene, were the main components in *Eucalyptus grandis*, and *α*-pinene, *γ*-terpinene, and *p*-cymene in *E. citriodora*. These EOs were found to dose-dependently inhibit germination of *Lactuca sativa*, with a concentration of 0.1 μL mL^−1^ of both oils suppressing germination by 74% and 68%, respectively [33].

With a preponderance of the monoterpenoids *p*-cymene, *β*-myrcene, and (+)-limonene, along with acenaphthene, EO extracted from *Artemisia scoparia* was found to have significant phytotoxic activities, primarliy against the roots of *Achyranthes aspera*, *Cassia occidentalis*, *Echinochloa crus-galli*, *Ageratum conyzoides*, and *Parthenium hysterophorus* [32] with the latter species suffering the most effects [32]. In the same manner, an artemisia ketone–rich EO derived from *Eucalyptus africanus* reportedly exhibited a potent effect similar to that *of Eucalyptus camaldulensis* on *Amaranthus hybridus*, but without a noticeable effect on *Portulaca oleracea* [23]. 

The phytotoxic activity of the EO of the Turkish *Origanum acutidens* and its monoterpenoid components (carvacrol, *p*-cymene, and thymol) were studied by Kordali, et al. [22]. Their results revealed that carvacrol and thymol completely inhibited seed germination and seedling growth in *Chenopodium album*, *Amaranthus retroflexus*, and *Rumex crispus*, but no effect was observed with *p*-cymene [22]. The EO of *Cymbopogon citratus*, which is composed mostly of the monoterpenes neral, geranial, and *β*-myrcene, was reported to delay the germination of seeds and inhibit seedling growth in *Echinochloa crus-galli* [21]. In another study, the EOs of the aerial parts of four plants—*Cymbopogon citratus*, *Origanum vulgare*, *Eucalyptus cladocalyx*, and *Artemisia absinthium*—were determined to be potential bioherbicides against the seeds of *Sinapis arvensis*, with the EOs of *Cymbopogon citratus* and *Eucalyptus cladocalyx* [20] exhibiting the most activity. Neral, geranial, and *α*-pinene, along with other monoterpenes, were found to be the main components of *Cymbopogon citratus*. However, the sesquiterpene spathulenol, as well as the monoterpenes 1,8-cineole and *p*-cymene, were found to be the main components of the EO of *Eucalyptus cladocalyx*. The three monoterpenoids carvacrol, *γ*-terpinene, and *p*-cymene were described as major constituents of the EO of *Origanum vulgare*, while *Artemisia absinthium* EO was reported to be composed largely of monoterpenes, including *β*-thujone, chamazulene, and linalool [20].

Potential herbicidal activity of the EO from *Nepeta flavida* was reported against *Raphanus sativus*, *Lepidium sativum*, and *Eruca sativa*, in which it completely inhibited germination at a concentration of 4.0 μL mL^−1^ [28], an effect that may be attributed to the presence of the monoterpenes linalool, 1,8-cineole, and sabinene [28]. Monoterpenes including carvacrol, *γ*-terpinene, *p*-cymene were found to be the predominant constituents of the EO of *Thymbra spicata* and may be responsible for the strong phytotoxic activity reported against *Zea mays*, *Triticum aestivum*, *Lactuca sativa*, *Lepidium sativum*, and *Portulaca oleracea* [27]. Ulukanli, et al. [26] reported that the EO of *Thymus eigii* exhibited significant toxic effects against *Lepidium sativum*, *Lactuca sativa*, and *P. oleracea*. This oil was found to be rich with monoterpenes, with thymol, carvacrol, and *p*-cymene the major constituents [26]. Moreover, EOs extracted from *Thymus daenensis* collected from four different habitats inhibited germination in *Avena fatua*, *Amaranthus retroflexus*, *Datura stramonium*, and *Lepidium sativum*. These four ecospecies of *Thymus daenensis* were found to be rich with monoterpenoids, including thymol, carvacrol, and *p*-cymene in particular [25].

Foliar volatiles of *Callistemon viminalis* and EOs reportedly reduced seed germination, seedling growth, and accumulation of dry matter in *Bidens pilosa*, *Cassia occidentalis*, *Echinochloa crusgalli*, and *Phalaris minor*, with the greatest sensitivity observed in *B. pilosa* [19]. Monoterpenoids, and 1,8-cineole, *α*-pinene, and *d*-limonene in particular, have been described as major components in the EO of this plant. Pinheiro, et al. [17] reported an allelopathic effect of the EO extracted from *Cannabis sativa* on germination and seedling growth in *Amaranthus retroflexus*, *Bromus secalinus*, *Avena sativa*, and *Brassica oleracea*. Based on gas chromatography-mass spectroscopy analysis, this oil is rich in monoterpenes, including myrcene, terpinolene, and (E)-*β*-Ocimene [18].

Monoterpene-rich EOs derived from *Schinus terebinthifolius* collected from two different areas of Brazil reportedly produced an inhibitory effect on germination, root and hypocotyl growth, and production of biomass in *Bidens pilosa*. In the Cerrado biome, the EO of *Schinus terebinthifolius* was found to be rich with *trans*-caryophyllene, 3-carene, and germacrene B, while *Schinus terebinthifolius* from the country’s Atlantic forest biome is rich with *α*-pinene, limonene, and *β*-pinene [17]. Additionally, the EO of *Vitex agnus-castus* was reported to be rich with monoterpenes, particularly 1,8-cineole, sabinene, *trans-β*-farnesene, and *α*-pinene. This EO was found to exhibit significant inhibitory activity on *Lactuca sativa* and *Lepidium sativum* [24].

The EO of *Salvia sclarea* was described to have significant phytotoxic effects against *Lepidium sativum*, *Lactuca sativa*, and *Portulaca oleracea* at a concentration of 0.16 mg mL^−1^, reducing seed germination by 94%, 100%, and 50%, respectively [16]. The main constituent of this EO was reported to be monoterpenes, including L-linalool, linalyl acetate, *α*-terpineol, and geraniol [16].

The EO from the aerial parts of *Euphorbia heterophylla* reportedly inhibited germination (93.9%), root (84.6%), and shoot growth (57.8%) in *Cenchrus echinatus* weeds at 100 μL L^−1^. The authors described monoterpenes as the major components (69.48 %), and 1,8-cineole was the primary monoterpene, representing 32.03% of the total mass [6].

## 4. Monoterpene-Rich EO-Allelopathy Correlation

**Figure 1:**
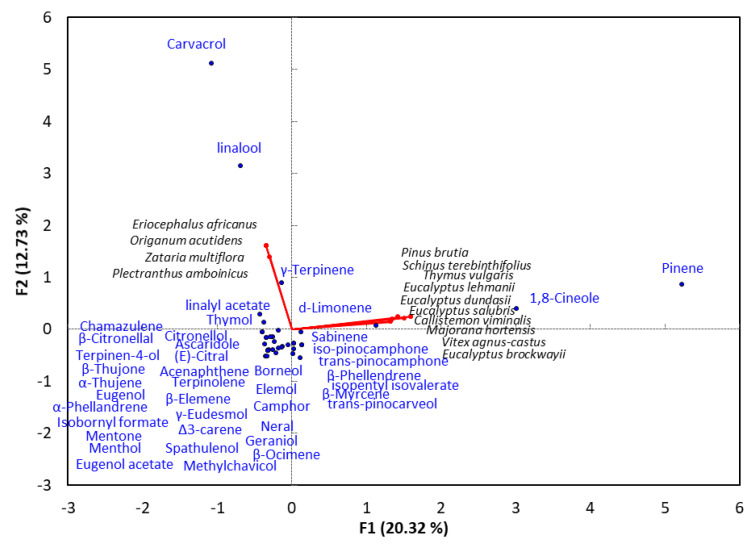
Principal component analysis of reported plants with essential oils containing monoterpenes as major compounds and showing allelopathic activity.

Application of a PCA to a dataset of the 46 different plant species with EOs comprised mainly of monoterpenes found allelopathic activity is presented in Figure 1. The results show that that *α*- and *β*-pinene, 1,8 cineole, linalool, and carvacrol were the most effective allelopathic monoterpene compounds. They also showed that *Eucalyptus africanus*, *Origanum acutidens*, *Zataria multiflora*, and *Plectranthus amboinicus* were correlated to each other linalool and carvacrol predominating (Figure 1). Meanwhile, *Pinus brutia*, *Schinus terebinthifolius*, *Thymus vulgaris*, *Eucalyptus lehmanii*, *Eucalyptus lehmanii*, *Eucalyptus lehmanii*, *Callistemon viminalis*, *Majorana hortensis*, *Vitex agnus-castus*, and *Eucalyptus brockwayii* showed a close correlation with each other with respect to the composition of their EOs; 1,8-cineole and *α*, and *β*-pinene were the major monoterpenoid compounds. The analysis found *α*-, and *β*-pinene and 1,8 cineole in most of the allelopathic plants in which monoterpenes are major EO compounds.

## 5. Phytotoxic EOs Derived from Plants Rich in Sesquiterpenes

Sesquiterpene-rich EOs from different plants associated with notable phytotoxic activities are listed in Table 2. The EO of *Eupatorium adenophorum* was described as being composed primarily of sesquiterpenes, with *γ*-cadinene, *γ*-muurolene, and 3-acetoxyamorpha-4,7(11)-diene-8-one as the main compounds. This oil reportedly exhibited strong phytotoxic activity against *Phalaris minor* and *Triticum aestivum*, with a stronger effect observed against *Phalaris minor* [48]. Elshamy and his co-workers reported the EO composition and allelopathic activities of three *Launaea* plants (*Launaea mucronata*, *Launaea nudicaulis*, and *Launaea spinosa*) collected from different habitats. Results showed that these EOs had significant and concentration-dependent effects on *Portulaca oleracea* weeds. The EOs of two samples of *Launaea mucronata* collected from the desert and coastal regions were found to have the highest activity, inhibiting germination by 96.1% and 87.9% and radicle growth by 92.6% and 89.7%, respectively, at 250 μL L^−1^ [6]. The authors found that sesquiterpenes were the main components, and hexahydrofarnesyl acetone the main compound, in *Launaea mucronata* [6]. The EO of *Schinus lentiscifolius* was reported to be associated with a 19.35% reduction in the mitotic index in onions and 25.14% in lettuce, compared with negative control. This EO was found to comprise sesquiterpenoid compounds as the main components, and *δ*-cadinene in particular [49].

A study of the chemical profiles of EOs of Tunisian *Ailanthus altissima* [53] deduced the presence of a high concentration of sesquiterpenes such as *β*-caryophyllene, (Z)-caryophyllene, germacrene D, and hexahydrofarnesyl acetone. The phytotoxic activities of the EOs (at a concentration of 1 mg mL^−1^) of the roots, stems, leaves, flowers, and fruits completely inhibited seed germination in *Lactuca sativa* [53]. *Raphanus sativus* and *Lepidium sativum* root growth was reduced under the effects of the EOs of *Nepeta curviflora* and *Nepeta nuda*. These EOs were found to have sesquiterpenes, and *β*-caryophyllene, caryophyllene oxide, and *β*-bisabolene in particular, as the main components. [31]. In addition, the EOs of *Teucrium maghrebinum*, *Teucrium polium*, and *Teucrium montbretii* were reported to be rich sources of sesquiterpenes, including caryophyllene, caryophyllene oxide, and carvacrol in particular. These EOs were found to significantly reduce the radicle growth of *Raphanus sativus* and *Lepidium sativum* with mild effect on germination [55].

The herbicidal effects of the EOs derived from *Lantana camara*, *Eucalyptus camaldulensis*, and *Eriocephalus africanus* were determined by Verdeguer, et al. [23]. The EO of *Eucalyptus camaldulensis*, which is reportedly composed primarily of spathulenol, had the greatest impact among the three plants, completely inhibiting seedling growth and germination in *Amaranthus hybridus* and *Portulaca oleracea*. With a high concentration of sesquiterpenes, and sesquiterpene hydrocarbons in particular. The EO of *Lantana camara* reportedly exhibited significant allelopathic activity against *Amaranthus hybridus* [23]. Recently, Elshamy, et al. [7] reported significant allelopathic effects of *Lactuca serriola* EO against *Bidens pilosa*, with half-maximal inhibitory concentrations (IC_50_) of 104.3, 92.3, and 140.3 μL L^−1^ for germination, growth of roots, and growth of shoots, respectively. The EO of *Lactuca serriola* was described to be rich with sesquiterpenes, with isoshyobunone and alloaromadendrene oxide-1 as major components. The EO of the invasive noxious plant *Heliotropium curassavicum*, collected from an inland area, demonstrated remarkable phytotoxic activities against *Chenopodium murale*, with IC_50_ values of 2.66, 0.59, and 0.70 mg mL^−1^ for germination, growth of roots, and growth of shoots, respectively. A coastal sample of the same species exhibited more allelopathic activity, with IC_50_ values of 1.58, 0.45, and 0.66 mg mL^−1^ [7]. Sesquiterpenes were determined to be the main class of EOs of *Heliotropium curassavicum*, and hexahydrofarnesyl acetone, (-)-caryophyllene oxide, and farnesyl acetone were the major compounds. In 2019, Abd El-Gawad and his co-authors reported that EOs from the leaves of the Egyptian *Xanthium strumarium* exhibited allelopathic effects against *Bidens pilosa*, and a concentration of 1000 µL L^−1^ inhibited seed, root, and shoot germination growth by 97.34%, 98.45%, and 93.56%, respectively [3]. In the EO of *Xanthium strumarium*, the sesquiterpenoids 1,5-dimethyltetralin, eudesmol, and l-borneol were the major identified compounds. 

The EO of *Symphyotrichum squamatum* collected from Egypt was analyzed and found to be enriched in sesquiterpenes such as humulene, epoxide, (-)-spathulenol, and (-)-caryophyllene oxide [15]. The EO of this plant was reported to have a strong and concentration-dependent allelopathic effect against *Bidens pilosa* weeds. The EO of *Cullen plicata*, rich in sesquiterpenes such as (−)-caryophylleneoxide, Z-nerolidol, tau cadinol, and *α*-cadinol, was reported to completely inhibit germination in *Bidens pilosa* and *Urospermum picroides* at 200 µL L_-1_ with respective IC_50_ values of 49.39 and 17.86 µL L^−1^ [50]. The EO derived from *Scutellaria strigillosa* was found to have significant phytotoxic potential against *Amaranthus retroflexus* and *Poa annua* [51]. These weeds were inhibited by 86.6 % and 20.0%, respectively, when treated with 1 µL ml^−1^ of *Scutellaria strigillosa* EOs. This active EO was found to be rich in sesquiterpenes, and germacrene D, 1-octen-3-ol, bicyclogermacrene, and *β*-caryophyllene in particular. In another study, the extracted EO of *Acroptilon repes* was examined by Razavi, et al. [52] for its phytotoxic activity against *Amaranthus retroflexus* and *Cardaria draba*. They reported that EOs from *Acroptilon repes* had a significant inhibitory effect on seed germination in *Amaranthus retroflexus*. Sesquiterpenes including caryophyllene oxide, *β*-cubebene, *β*-caeyophyllen, and *α*-copaen were reported as the main constituents of this EO [52].

Recently, Assaeed et al. reported that sesquiterpene-rich EOs of the aerial parts of *Pulicaria somalensis* had significant phytotoxic effects on the weeds of *Dactyloctenium aegyptium* and *Bidens poilosa*, with an IC_50_ of 0.6 mg mL^−1^ for root growth in both weeds, and 0.7 and 1.0 mg mL^−1^ for shoot growth, respectively. Juniper camphor (24.7%), *α*-sinensal (7.7%), 6-epi-shyobunol (6.6%), and *α*-zingiberene (5.8%) were reported to be the main chemical constituents of the EO of this plant [54]. 

Lastly, the EO of aboveground parts of *Bassia muricata* (Chenopodiaceae) was found to have a significant reduction effect on root growth, shoot growth, and germination in *Chenopodium murale* weed, with IC_50_ values of 175.60 µL L^−1^, 246.65 µL L^−1^, and 308.33 µL L^−1^, respectively. Sesquiterpenes were found to be the main constituents of the EO, with an abundance of hexahydrofarnesyl acetone, and *α*-gurjunene [54]. 

## 6. Sesquiterpene-Rich EO-Allelopathy Correlation

**Figure 2:**
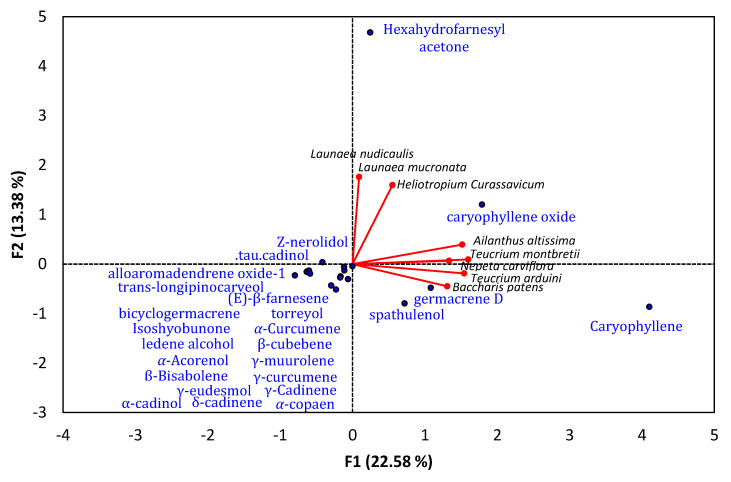
Principal component analysis of reported plants with essential oils containing sesquiterpenes as major compounds and showing allelopathic activity.

The application of PCA to a dataset of 15 different plant species with EOs composed mainly of sesquiterpenoid compounds showed allelopathic activity is presented in Figure 2. Caryophyllene, caryophyllene oxide, germacrene D, spathulenol, and hexahydrofarnesyl acetone were the sesquiterpenoids most associated with allelopathic activity. Most tested plant species were correlated to each other regarding these major EO compounds.

## 7. Phytotoxic EOs Derived from Plants Rich in Non-Terpenoids 

Phytotoxic EOs with non-terpenoid major compounds are listed in Table 3. The EOs of leaves and fruits of *Ecballium elaterium* reportedly contain phenolics and hydrocarbons, including E-anethol, octyl octanoate, 3-(6,6-dimethyl-5-oxohept-2-enyl)-cyclohexanone, and tetracosane as major components [56]. The EO of the leaves was found to have an allelopathic effect on *Lactuca sativa* that was stronger than that of fruits, with a significant (12%) decrease in seed germination. In another study, Mutlu, et al. [57] found that EO rich in iridoids from *Nepeta meyeri* had a strong inhibitory effect (>50%) on seed germination of *Bromus danthoniae*, *Bromus intermedius*, and *Lactuca serriola* at a concentration of 0.01% and 0.02%. Kordali, et al. [58] reported that the EO of the Turkish plant *Nepeta meyeri* contained 4a-*α*,7-*α*,7*a-β* nepetalactone and 4*a-α*,7-*α*,7*a-α* nepetalactone as major compounds. This EO completely inhibited germination of *Amaranthus retroflexus*, *Chenopodium album*, *Cirsium arvense*, and *Sinapsis arvensis* at a concentration of 0.5 mg mL^−1^. Iridoids, and 4a-*α*,7-*α*,7a-*β*-nepetalactone and 4a-*α*,7-*β*,7a-*α*-nepetalactone in particular, were determined to be the major compounds of the EO of *Nepeta cataria* [59]. This EO can reportedly act as an allelochemical agent against *Hordeum spontaneum*, *Taraxacum officinale*, *Avena fatua*, and *Lipidium sativum*, with dose-dependent suppression of germination [59]. Similarly, Bozok, et al. [60] reported a strong herbicidal activity for the EO of *Nepeta nuda* on germination and seedling growth in *Raphanus sativus*, *Triticum aestivum*, *Lactuca sativa*, *Portulaca oleracea*, and *Lepidium sativum*. This EO was rich in iridoids 4a-*α*,7-*α*,7*α*-*β*-nepetalactone, 2(1H)-naphthalenone, and trans-octahydro-8a-methyl. Finally, Mancini, et al. [31] reported that the EOs of the two Salvia species (*Salvia hierosolymitana* and *Salvia multicaulis*) displayed phytotoxic effects on *Raphanus sativus* by reducing radicle elongation and seed germination. These EOs are characterized by an abundance of carbonylic compounds.

## 8. Structure-Activity Relationship Summary

Based on the data presented in Table 1, Table 2 and Table 3 and correlation analysis between phytotoxic EOs derived from different plants and their major chemical constituents (Figure 1 and Figure 2), we concluded that the phytotoxic activities of EOs increase with terpenoid content, particularly oxygenated terpenoid content. Almost all previous studies found that increasing the oxygenation of terpenoids led to an increase in allopathic activities via inhibition of germination and growth of noxious weeds [6,16,23]. 

As can be seen in Table 1, oxygenated monoterpenoids are the main components of phytotoxic EOs, and their phytotoxicity was observed to increase with the degree of oxygenation. For example, the mono-oxygenated monoterpenoid 1,8-cineole (eucalyptol, C_10_H_18_O), was to be the main compound in several allopathic EOs derived from plants from different botanical families such as *Euphorbia heterophylla* [6], *Callistemon viminalis* [19], *Eucalyptus cladocalyx* [20], *Nepeta flavida* [28], *Majorana hortensis* [40], *Peumus boldus* [43], *Eucalyptus lehmanii* [45], *Tanacetum aucheranum*, *Tanacetum chiliophyllum* [46], *Eucalyptus salubris*, *Eucalyptus brockwayii*, and *Eucalyptus dundasii* [14]. 

In addition, linalool and borneol were found to be the major compounds in numerous phytotoxic oils, such as *Salvia sclarea* [16], *Artemisia absinthium* [20], *Origanum acutidens* [22], *Eriocephalus africanus* [23], *Nepeta flavida* [28], *Zataria multiflora* [38], *Agastache rugosa* [44], *Salvia officinalis*, and *Ocimum basilicum* [40], and *Tanacetum chiliophyllum* [46]. It is therefore clear that the oxygenated monoterpenoids 1,8-cineole, linalool, and borneol play significant and effective roles as allopathic agents and more research into their phytotoxic activity and phytotoxic mechanism(s) is recommended. 

Similarly, careful analysis of sesquiterpene-rich phytotoxic EOs revealed that an increase in oxygenated sesquiterpene levels can enhance the phytotoxic activity of an EO. The data supplied in Table 2 and PCA analysis suggest the major oxygenated sesquiterpenes caryophyllene and its derivatives, as well as hexahydrofarnesyl acetone, can be potent phytotoxic agents. The phytotoxic EOs derived from *Baccharis patens* [29,30], *Heliotropium curassavicum* [7], *Cullen plicata* [50], *Scutellaria strigillosa* [51], *Acroptilon repens* [52], *Lantana camara* [23], *Teucrium arduini*, *Teucrium maghrebinum*, *Teucrium polium*, *Teucrium montbretii* [31], and *Ailanthus altissima* [53] were reported to have all or one of *β*-caryophyllene, (-)-caryophyllene, and caryophyllene oxide as primary compounds. These reports indicate a strong correlation between the phytotoxic activities of EOs and the presence of these compounds as main components. Hexahydrofarnesyl acetone has been described as an essential compound in the phytotoxic EOs of *Launaea mucronata*, *Launaea nudicaulis* [6], *Heliotropium curassavicum* [7], and *Ailanthus altissima* [53]. The authors of these studies also concluded that compounds with hexahydrofarnesyl acetone as a main constituent can play a major role as phytotoxic mediators.

EOs derived from aromatic plants typically consist of low-molecular-weight terpenoids, including mono, sesqui-, and diterpenoids as well as non-terpenoid components [14]. Two plants belonging to the *Nepeta* genus were reported that containing iridoid-rich EOs such as *Nepeta meyeri* [57,58] and *Nepeta cataria* [59]. The two iridoids 4a-*α*,7-*α*,7a-*β*-nepetalactone and 4a-*α*,7-*β*,7a-*α*-nepetalactone were reported to be the main phytotoxic mediators in the EOs of these two species. The two compounds should, therefore, be subjected to further study to evaluate their allopathic abilities against several noxious weeds.

## Figures and Tables

**Table 1:** Monoterpene-rich EOs derived from various reported plants with significant allelopathic activity.

Plant Name	Main Monoterpenoid Compounds	Phytotoxic against	Reference
*Euphorbia heterophylla*	1,8-cineole, camphor,	*Cenchrus echinatus* *	[6]
*Symphyotrichum squamatum*	*β*-pinene	*Bidens pilosa* *	[15]
*Salvia sclarea*	l-linalool, linalyl acetate, *α*-terpineol, and geraniol	*Lactuca sativa*, *Lepidium sativum*, and *Portulaca oleracea* *	[16]
*Schinus terebinthifolius*	3-carene, *α*-pinene, limonene, and *β*-pinene	*Bidens pilosa* *	[17]
*Cannabis sativa*	myrcene, terpinolene, and (*E*)-*β*-ocimene	*Avena sativa*, *Zea mays*, *Brassica oleracea*, *Avena fatua* *, *Bromus* *secalinus* *, *Echinochloa* *crus-galli* *, *Amaranthus retroflexus* *, *Centaurea cyanus* *	[18]
*Callistemon viminalis*	1,8-cineole *α*-pinene, and d-limonene	*Bidens pilosa* *, *Cassia occidentalis* *, *Echinochloa crus-galli* *, and *Phalaris minor* *	[19]
*Cymbopogon citratus*	neral, geranial, and *α*-pinene	*Sinapis arvensis* *	[20]
*Eucalyptus cladocalyx*	1.8-cineole, and *p*-cymene		
*Origanum vulgare*	carvacrol, *γ*-terpinene, and *p-*cymene		
*Artemisia absinthium*	*β*-thujone, and linalool		
*Cymbopogon citratus*	neral, geranial, and *β*-myrcene	*Echinochloa crus-galli* *	[21]
*Origanum acutidens*	carvacrol, *p-*cymene, linalool acetate	*Amaranthus retroflexus* *, *Chenopodium album* *, and *Rumex crispus* *	[22]
*Eriocephalus* *africanus*	carvacrol, *p-*cymene, linalool acetate	*Amaranthus hybridus* * and *Portulaca oleracea* *	[23]
*Vitex agnus-castus*	1,8-cineole, sabinene, and *α*-pinene	*Lactuca sativa* and *Lepidium sativum*	[24]
*Thymus daenensis*	thymol, carvacrol, and *p-*cymene	*Amaranthus* *retroflexus* *, *Avena fatua* *, *Datura stramonium* *, and *Lepidium sativum*	[25]
*Thymus eigii*	thymol, carvacrol, *p-*cymene, *γ*-terpinene, and borneol	*Lactuca sativa*, *Lepidium sativum*, and *Portulaca oleracea* *	[26]
*Thymbra spicata*	carvacrol, *γ*-terpinene, *p-*cymene	*Triticum aestivum*, *Zea mays*, *Lactuca sativa*, *Lepidium sativum*, and *Portulaca oleracea* *	[27]
*Nepeta flavida*	linalool, 1,8-cineole, and sabinene	*Lepidium sativum*, *Raphanus sativus*, and *Eruca sativa*	[28]
*Heterothalamus psiadioides*	*β*-pinene, *δ*^3^-carene, and limonene	*Lactuca sativa* and *Allium cepa*	[29,30]
*Salvia hierosolymitana*	*α*-pinene, myrtenol, and sabinyl acetate,	*Raphanus sativus* and *Lepidium* *sativum*	[31]
*Artemisia scoparia*	*p-*cymene, *β*-myrcene, and (+)-limonene	*Achyranthes aspera*, *Cassia occidentalis* *, *Parthenium hysterophorus* *, *Echinochloa crus-galli* *, and *Ageratum conyzoides* *	[32]
*Eucalyptus grandis*	*α*-pinene, *γ*-terpinene, and *p-*cymene	*Lactuca sativa*	[33]
*Eucalyptus citriodora*	*β*-citronellal, geraniol, and citronellol		
*Plectranthus* *amboinicus*	carvacrol	*Lactuca sativa* and *Sorghum bicolor*	[34]
*Pinus brutia*	*α*-pinene, and *β*-pinene	*Lactuca sativa*, *Lepidium sativum*, and *Portulaca oleracea* *	[35]
*Pinus pinea*	limonene, *α*-pinene, and *β*-pinene	*Sinapis arvensis* *, *Lolium* *rigidum* *, and *Raphanus raphanistrum* *	[36]
*Cotinus coggyria* Scop.	*α*-pinene, limonene, and *β*-myrcene	*Silybum marianum* *, and *Portulaca oleracea* *	[37]
*Zataria* *multiflora*	carvacrol, linalool and *p-*cymene	*Hordeum spontaneum* *, *Secale cereale* *, and *Amaranthus retroflexus* *, and *Cynodon dactylon* *	[38]
*Mentha × piperita*	menthol, mentone, and menthofuran	*Lycopersicon esculentum*, *Raphanus sativus*, *Convolvulus arvensis* *, *Portulaca oleracea* *, and *Echinochloa colonum* *	[39]
*Hyssopus officinalis*	*β*-pinene, iso-pinocamphone, and, *trans*-pinocamphone	*Raphanus sativus*, *Lactuca sativa*, and *Lepidium sativum*	[40]
*Lavandula angustifolia*	*β*-pinene, iso-pinocamphone, and *trans*-pinocamphone		
*Majorana hortensis*	1,8-cineole, *β*-phellandrene, and *α*-pinene		
*Melissa officinalis*	(-)-citronellal, carvacrol, and citronellol		
*Ocimum basilicum*	linalol, and borneol		
*Origanum vulgare*	*o*-cymene, carvacrol, and linalyl acetate		
*Salvia officinalis*	*trans*-thujone, camphor, and borneol		
*Thymus vulgaris*	*o*-cymene, and *α*-pinene		
*Verbena officinalis*	isobornyl formate , and (*E*)-citral		
*Shinus molle*	*β*-phellendrene, *α*-phellendrene, and myrcene	*Triticum aestivum*	[41]
*Syzygium aromaticum*	eugenol, and eugenol acetate	*Mimosa pudic* *a and *Senna obtusifolia* *	[42]
*Peumus boldus*	ascaridole, *p-*cymene and 1,8-cineole	*Amaranthus hybridus* * and *Portulaca oleracea* *	[43]
*Drimys winterii*	terpinen-4-ol, γ-terpinene, and sabinene		
*Agastache rugosa*	*d*-limonene, and linalool	*Majorana hortensis* *, *Trifolium repens* *, *Rudbeckia hirta*, *Chrysanthemum zawadskii*, *Melissa officinalis* *, *Taraxacum platycarpum* *, and *Tagetes patula*	[44]
*Eucalyptus lehmanii*	1,8-cineole, *α*-thujene, and *α*-pinene	*Sinapis arvensis* *, *Diplotaxis harra* *, *Trifolium campestre* *, *Desmazeria rigida* *, and *Phalaris canariensis* *	[45]
*Tanacetum aucheranum*	1,8-cineole, camphor, and terpinen-4-ol	*Amaranthus retroflexus* *, *Chenopodium album* *, and *Rumex crispus* *	[46]
*Tanacetum chiliophyllum*	camphor, 1,8-cineole and borneol		
*Heterothalamus psiadioides*	*β*-pinene, *δ*^3^-carene, and limonene	*Lactuca sativa* and *Allium cepa*	[29]
*Baccharis patens*	linalool		
*Senecio amplexicaulis*	*α*-phellandrene, *o*-cymene and *β*-ocimene	*Phalaris minor* * and *Triticum aestivum*	[47]
*Eucalyptus salubris*	1,8-cineole, *α*-pinene and *p-*cymene	*Solanum elaeagnifolium* *	[14]
*Eucalyptus dundasii*	1,8-cineole, *α*-pinene and trans-pinocarveol		
*Eucalyptus spathulata*	1,8-cineole and *α*-pinene		
*Eucalyptus brockwayii*	*α*-pinene, 1,8-cineole and isopentyl isovalerate		
*Carum carvi*	estragole, limonene, and *β*-pinene	*Raphanus sativus*, *Lactuca sativa*, and *Lepidium sativum*	[40]

* Reported as a weed.

**Table 2:** Sesquiterpene-rich EOs derived from various plants and exhibiting phytotoxic activity.

Plant Name	Major Sesquiterpenes Compounds	Phytotoxic Against	Reference
*Lactuca serriola*	isoshyobunone, and alloaromadendrene oxide-1	*Bidens pilosa* *	[7]
*Launaea mucronata*	hexahydrofarnesyl acetone and (-)-spathulenol	*Portulaca oleracea* *	[6]
*Launaea nudicaulis*	hexahydrofarnesyl acetone and *γ*-gurjunen epoxide (2)		
*Launaea spinosa*	*α*-acorenol, trans-longipinocarveol, and *γ*-eudesmol		
*Heliotropium curassavicum*	Hexahydrofarnesyl acetone, (-)-caryophyllene oxide, farnesyl acetone	*Chenopodium murale* *	[6]
*Xanthium strumarium*	*α*-eudesmol, (-)-spathulenol, and ledene alcohol	*Bidens pilosa* *	[3]
*Cullen plicata*	(−)-caryophyllene oxide, z-nerolidol, tau.cadinol and *α*-cadinol	*Bidens pilosa* * and *Urospermum picroides* *	[50]
*Scutellaria strigillosa*	germacrene D, bicyclogermacrene, and *β*-caryophyllene	*Amaranthus retroflexus* * and *Poa annua* *	[51]
*Acroptilon repens*	caryophyllene oxide, *β*-cubebene, *β*-caeyophyllen, and *α*-copaen	*Amaranthus* *retroflexus* * and *Cardaria draba* *	[52]
*Lantana camara*	*α*-curcumene, *β*-caryophyllene, and *γ*-curcumene	*Amaranthus hybridus* * and *Portulaca oleracea* *	[23]
*Eucalyptus camaldulensis*	spathulenol, and isobicyclogermacrenal		
*Eupatorium adenophorum*	*γ*-cadinene, *γ*-muurolene, and 3-acetoxyamorpha-4,7(11)-diene-8-one	*Phalaris minor* * and *Triticum aestivum* *	
*Baccharis patens*	*β*-caryophyllene, and spathulenol	*Lactuca sativa* and *Allium cepa*	[29]
*Salvia multicaulis*	*α*-Copaene, *β*-caryophyllene, and aromadendrene	*Raphanus sativus* and *Lepidium* *sativum*	[40]
*Teucrium arduini*	caryophyllene, caryophyllene oxide, germacrene D, and spathulenol		
*Teucrium maghrebinum*	germacrene d, *δ*-cadinene, *γ*-cadinene, and caryophyllene		
*Teucrium polium*	caryophyllene, torreyol, and *α*-cadinol		
*Teucrium montbretii*	carvacrol, caryophyllene, and caryophyllene oxide		
*Nepeta curviflora*	*β*-caryophyllene, caryophyllene oxide, and (*E*)-*β*-farnesene		[31]
*Nepeta nuda*	*β*-bisabolene		
*Ailanthus altissima*	*β*-caryophyllene, (Z)-caryophyllene, and germacrene D,	*Lactuca sativa*	[53]
*Schinus lentiscifolius*	δ-cadinene, *α*-cadinol, and *β*-caryophyllene		[49]
*Pulicaria somalensis*	Juniper camphor (24.7%), *α*-sinensal (7.7%), 6-epi-shyobunol (6.6%), and *α*-zingiberene (5.8%)	*Dactyloctenium aegyptium* * and *Bidens pilosa* *	[4]
*Bassia muricata*	hexahydrofarnesyl acetone, and *α*-gurjunene	*Chenopodium murale* *	[54]

* Reported as a weed.

**Table 3:** Nonterpenoidial-rich EOs derived from plants and with significant phytotoxic activity.

Plant Name	Main Components	Major Compounds	Phytotoxic Against	Reference
*Nepeta nuda*	Iridoids	4a-*α*,7-*α*,7a*-β*-nepetalactone, 2(1h)-naphthalenone, and octahydro-8a-methyl-trans-	*Triticum aestivum*, *Raphanus sativus*, *Lactuca sativa*, *Lepidium sativum*, and *Portulaca oleracea* *	[60]
*Nepeta cataria*	Iridoids	4a-*α*,7-*α*,7a-*β*-nepetalactone and 4a-*α*,7-*β*,7a-*α*-nepetalactone	*Hordeum spontaneum* *, *Taraxacum officinale* *, *Avena fatua* *, and *Lipidium sativum*	[59]
*Nepeta meyeri*	Iridoids	4a-*α*,7-*α*,7a-*β*-nepetalactone and 4a-*α*,7-*β*,7a-*α*-nepetalactone	*Amaranthus* *retroflexus* *, *Bromus danthoniae* *, *Bromus* *intermedius* *, *Chenopodium album* *, *Cynodon dactylon* *, *Lactuca serriola* *, *Portulaca oleracea* *, *Cirsium arvense* *, and *Sinapsis arvensis* *	[57,58]
*Ecballium* *elaterium*	Phenolics and hydrocarbons	*e*-anethol, octyl octanoate, 3-(6,6-dimethyl-5-oxohept-2-enyl)-cyclohexanone, and tetracosane	*Lactuca sativa*	[56]
*Pimpinella anisum*	Non-terpenoidial phenols	*cis*-anethole	*Raphanus sativus*, *Lactuca sativa*, and *Lepidium sativum*	[40]
*Foeniculum* *vulgare*	Non-terpenoidial phenols	*cis*-anethole		

* Reported as a weed.
